# Dilated cardiomyopathy in Rubinstein-Taybi syndrome: A case report and mini-review of the literature

**DOI:** 10.3892/mi.2025.281

**Published:** 2025-10-29

**Authors:** Ahsen Shah, Asad Riaz, Abdul Muhymin Alam Khattak, Hasham Qureshi, Zanib Ejaz, Muhammad Mustafa

**Affiliations:** 1 Department of General Medicine, Ayub Teaching Hospital, Abbottabad 22010, Pakistan; 2 Department of General Surgery, Ayub Teaching Hospital, Abbottabad 22010, Pakistan; 3 Department of Medical Oncology, St. Vincent's Private Hospital, Dublin 4, D04 N2E0, Ireland; 4 North Dublin Mental Health Service, Dublin D09 X85P, Ireland

**Keywords:** Rubinstein-Taybi syndrome, dilated cardiomyopathy, genetic mutations, congenital heart defects

## Abstract

Rubinstein-Taybi syndrome (RTS) or Broad Thumb-Hallux syndrome, is a rare neurodevelopmental disorder characterized by distinctive physical, cognitive and congenital abnormalities. Mutations in the CREBBP or EP300 genes are implicated, often arising *de novo*. While cardiac defects are noted in 32.6% of patients with RTS, the association with dilated cardiomyopathy (DCM) remains poorly explored. The present study describes a clinically diagnosed case of a 32-year-old male patient with RTS, manifesting with symptoms of heart failure. The patient, born to a consanguineous marriage, exhibited hallmark features of RTS, including short stature, dysmorphic facial features, intellectual disability and broad thumbs. DCM was confirmed by echocardiography with an ejection fraction of 20%. The patient responded well to diuretics for heart failure and was referred for specialized cardiology and surgical management. Cardiac manifestations in RTS vary from septal defects to complex anomalies, with few reports on DCM. The genetic basis of RTS may contribute to cardiac dysfunction, underscoring the need for multidisciplinary care. The case described herein highlights the necessity of recognizing RTS in adults with unexplained syndromic features and cardiac symptoms. Comprehensive evaluation, including cardiac screening, is essential for improving patient outcomes. Further research is warranted to establish the link between RTS and DCM and to develop diagnostic and therapeutic guidelines.

## Introduction

Rubinstein-Taybi syndrome (RTS), also known as Broad Thumb-Hallux syndrome, was initially described by Michail *et al* in 1957(1) and further characterized by Rubinstein and Taybi in 1963(2). This is a rare neurodevelopmental disorder with an incidence of 1 in 125,000 individuals and is characterized by wide spectrum of features, such as a short stature, broad thumbs and halluces, facial abnormalities (downslanted palpebral fissures, arched/thick eyebrows, beaked nose with a hanging columella below the nostrils, microtia and low set ears, micrognathia, ptosis, a high arched palate, cleft lip/palate, atypical/grimacing smile, overcrowded teeth and talon cusps, microcephaly), postnatal growth delay and intellectual disability (3,4). Other common associated findings in RTS are described in Table I (5).

Although the exact underlying pathology remains unclear, RTS is considered to be caused by mutations in the cyclic-AMP-regulated enhancer binding protein (CREBBP) gene on chromosome 16 and, less frequently, in the E1A binding protein p300 (EP300) gene on chromosome 22 (5,6). These mutations are typically *de novo* with a minimal chance of transmission from parents to the next generation (7). The syndrome usually requires management through a multidisciplinary approach due to its various health-related implications. Affected individuals often require proper care throughout their lives. The present study describes the first case of a clinically diagnosed 32-year-old male patient with RTS in Pakistan, presenting with features of heart failure.

## Case report

A 32-year-old male patient, born out of a consanguineous marriage with no family history of RTS, presented to the Outpatient Department of Ayub Teaching Hospital, Abbottabad, Pakistan, with chief complaints of abdominal distension, shortness of breath and generalized swelling. These symptoms developed gradually over a few months. The case history provided by the attendant revealed that the patient had been intellectually disabled and deaf-mute since birth.

### Clinical examination. General condition of the patient

The patient had a short stature with a syndromic face. The patient also had intellectual disability and congenital deaf-mutism.

*Cardiovascular condition*. The patient was found to have raised jugular venous pressure, a displaced apex beat to the left and high-grade pansystolic murmur.

*Abdominal findings*. The patient exhibited gross distension with positive shifting dullness and fluid thrill, no palpable visceromegaly, bilateral pitting pedal edema up to the knees and normal vital signs.

### Diagnostic criteria and features of RTS

**Figure 1:**
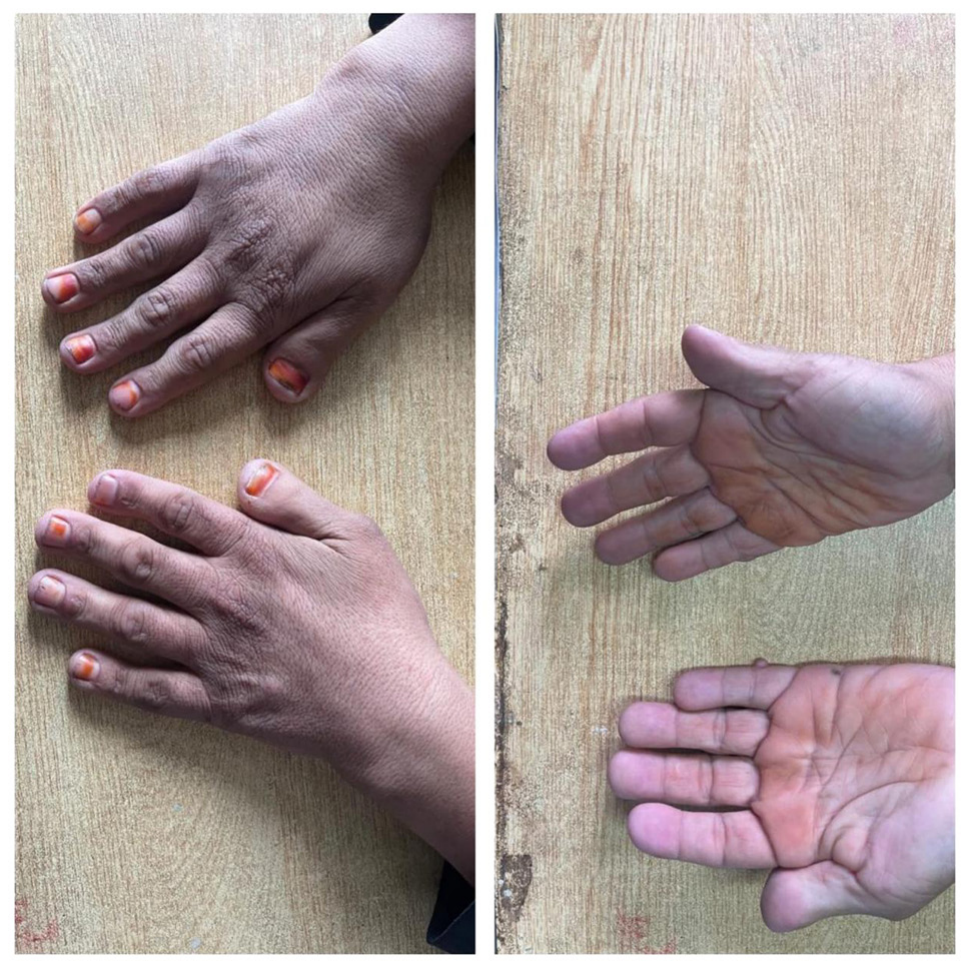
Characteristic hand anomalies in Rubinstein-Taybi syndrome. Dorsal and palmar views of both hands illustrating broad, angulated thumbs, shortened fingers and broad terminal phalanges.

**Figure 2:**
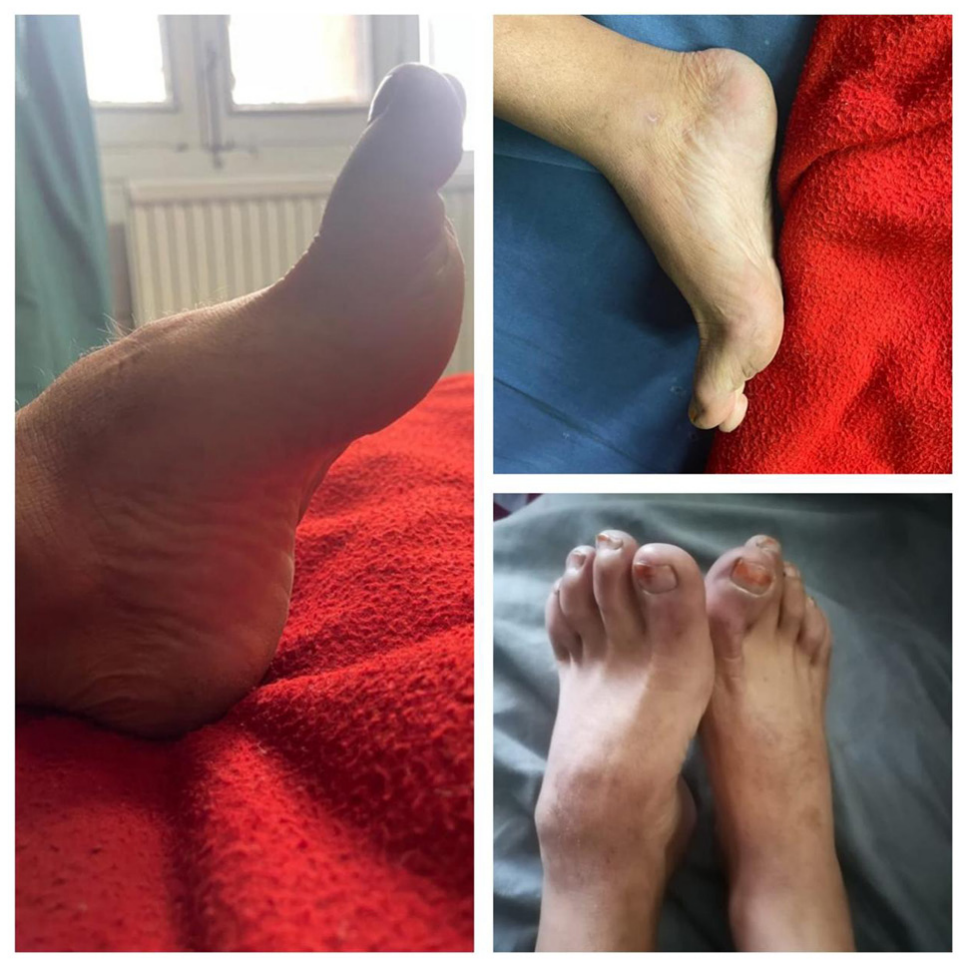
Foot abnormalities in Rubinstein-Taybi syndrome. Broad and medially deviated halluces, short broad toes with nail changes and pes cavus deformity are demonstrated.

The clinical diagnosis of RTS should adhere to the consensus criteria established by Lacombe *et al* (8). The criteria for RTS are as follows, which were met by the patient in the present study (apart from genetic testing):

*Essential criteria*: Intellectual disability, broad and angulated thumbs/halluces (Figs. 1 and 2), and pathogenic CREBBP or EP300 mutation (if confirmed by genetic testing).

**Figure 3:**
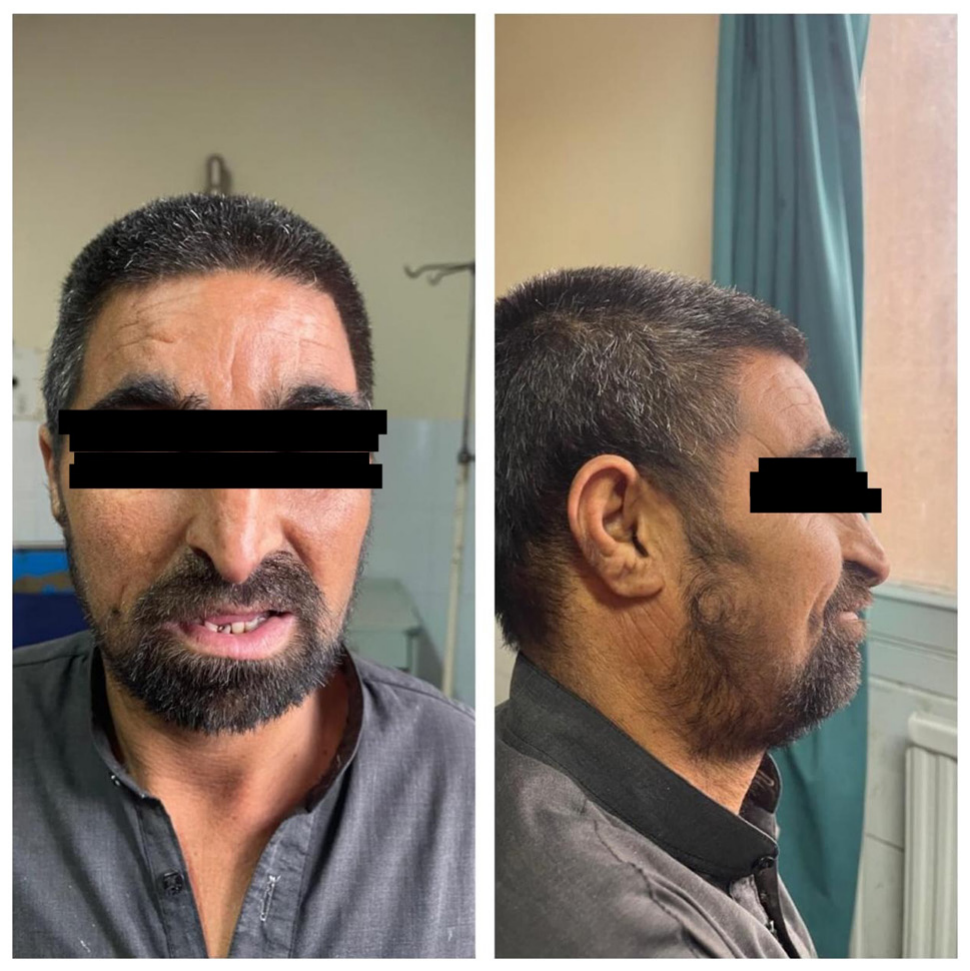
Facial features in Rubinstein-Taybi syndrome. Frontal and lateral views illustrating prominent nasal bridge, a beaked nose, thick and arched eyebrows, hypertelorism and mild mandibular asymmetry.

**Figure 4:**
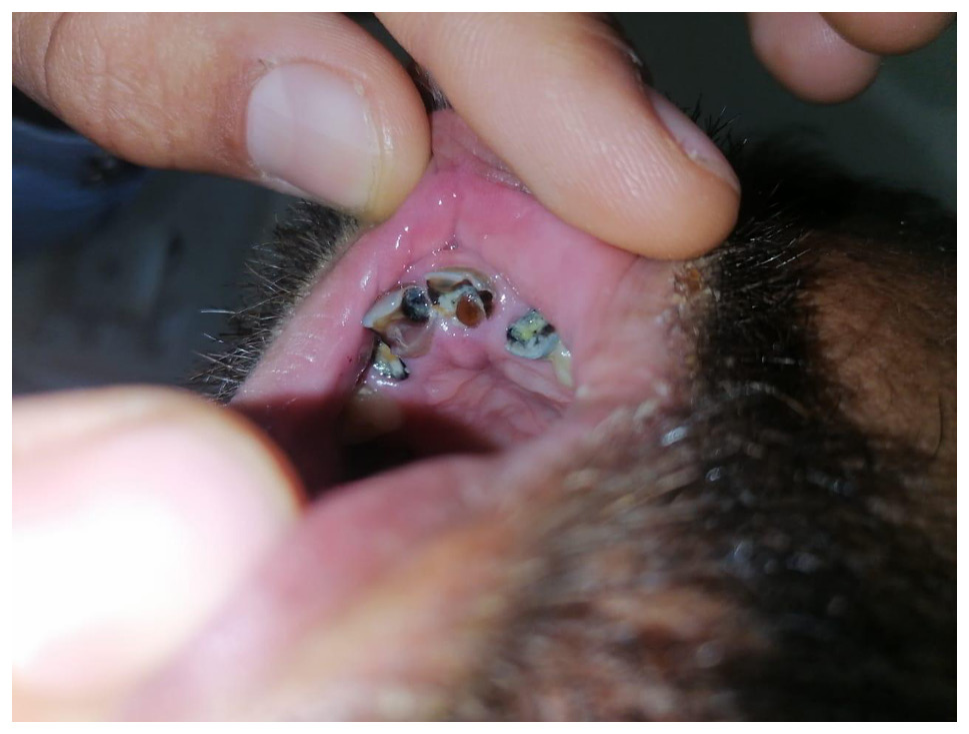
Severe dental caries in a patient with Rubinstein-Taybi syndrome. Clinical image illustrating multiple posterior teeth with extensive crown destruction, secondary caries around previous restorations and poor oral hygiene.

*Major criteria*: Characteristic craniofacial dysmorphism such as thick/arched eyebrows, hypertelorism, downslanting palpebral fissures, beaked nose and grimacing smile (Fig. 3), dental anomalies such as talon cusps, high-arched palate, micrognathia, malocclusion (crowding, anterior open bite), hypodontia, microdontia, delayed eruption, narrow dental arches and increased susceptibility to dental caries (Fig. 4) and cryptorchidism (in males).

*Minor criteria*: A short stature, microcephaly and congenital hearing impairment.

### Cardiac involvement: Dilated cardiomyopathy (DCM)

The patient was found to suffer from DCM. He had severe left ventricular dysfunction [left ventricular ejection fraction (LVEF) of 20%], mitral regurgitation, dilated ventricles with global hypokinesis, an increased left ventricular end-diastolic diameter (70 mm), an increased left ventricular end-systolic diameter (60 mm), normal interventricular septal thickness (10 mm), left atrial enlargement (diameter, 45 mm) and no septal defects on echocardiography.

*Investigations and management*. Baseline investigations yielded normal findings, apart from a slightly increased alanine aminotransferase (ALT) level. The following laboratory investigations were performed for the patient: Electrolyte levels were normal (sodium, 140 mEq/l; potassium, 4.3 mEq/l; and chloride, 102 mEq/l). The serum albumin level was 3.6 g/dl, and the total bilirubin level was 1.8 mg/dl. Liver function tests (LFTs) revealed an alkaline phosphatase level of 98 U/l and an alanine aminotransferase (ALT) level of 31 U/l, both within normal limits apart from the mildly elevated bilirubin level.

Renal function tests (RFTs) revealed a blood urea nitrogen level of 15 mg/dl and serum creatinine level of 1.0 mg/dl, indicating normal renal function. Fluid R/E was unremarkable. The activated partial thromboplastin time (APTT) was 33 sec, and the PT/INR was 12 sec/1.0, both normal. Urinalysis with microscopy revealed a clear appearance with no protein, glucose, RBCs, or casts. The C-reactive protein (CRP) level was 3 mg/l, the phosphorus level was 3.7 mg/dl, the serum calcium level was 9.3 mg/dl, and the serum amylase level was 62 U/l. Overall, all other parameters were within normal limits, with only a mildly elevated total bilirubin and slightly low-normal albumin noted. Ascitic fluid analysis revealed a SAAG level >1.1, normal ADA levels. An echocardiography confirmed the presence of DCM with detailed measurements.

The patient was treated with intravenous furosemide (40 mg, twice daily), leading to significant diuresis, and the resolution of ascites and limb edema within 2 days. He exhibited clinical improvement with reduced dyspnea and an increased urine output. He was subsequently referred to a cardiologist for the further management of heart failure. A follow-up with a surgeon was also arranged to address cryptorchidism.

## Discussion

RTS is a genetic condition marked by distinct physical traits, cognitive impairment and various congenital abnormalities, including heart defects. DCM has been increasingly observed among the cardiac issues linked to RTS. In the present study, a review of the literature was also performed to compile existing cases of RTS with DCM, highlighting the range of clinical presentations, related heart anomalies and patient outcomes.

RTS results from mutations in either the CREBBP or EP300 genes, which play crucial roles in gene expression regulation and chromatin modification. The syndrome is typified by wide thumbs and toes, facial irregularities and varying levels of intellectual disability. Heart abnormalities are noted in approximately one-third of patients with RTS, often presenting as isolated septal defects or patent ductus arteriosus (PDA) (9). While less common, the occurrence of DCM in RTS has been documented, suggesting a need for a more in-depth exploration of the underlying mechanisms and treatment approaches. The review performed herein identified various cases of RTS associated with DCM and other cardiac abnormalities, including a newly reported case (Table I).

The identified cases demonstrate the diverse cardiac manifestations observed in patients with RTS. Some individuals exhibit simple defects such as PDA (Case 1, Table I) or ventricular septal defect (VSD) (Case 3, Table I), while others exhibit more intricate abnormalities, including tricuspid and pulmonary atresia (Case 2, Table I). In the present study, the newly reported case of a 32-year-old male patient with RTS and DCM sheds light on the adult presentation of this syndrome, which is less frequently documented. This patient experienced marked heart failure symptoms, such as abdominal swelling and dyspnea, requiring diuretic treatment.

The occurrence of DCM in patients with RTS indicates a possible connection between the genetic underpinnings of the syndrome and heart muscle function. The case described herein underscores the need to recognize RTS in adults, as its clinical manifestation may differ from that in children. The management of DCM in patients with RTS may be complex, often necessitating a collaborative approach involving pediatric cardiologists, geneticists and surgeons. Surgical procedures may be required for marked cardiac anomalies, while ongoing monitoring and supportive care are vital for addressing heart failure symptoms associated with DCM.

RTS is a rare congenital multiple anomaly syndrome. First described in 1957 by Michail *et al* (1), followed by Rubinstein and Taybi in 1963(2). The underlying cause is not known, although research has shown genetic mutations in the CREBBP gene and EP300 gene as a possible cause (5). The vast majority of cases occur due to spontaneous mutations with a rare chance of transmission from parents to offspring (7). There are no precise and defined diagnostic criteria for RTS; however, diagnosis can be made by genetic studies, although this is mostly performed based on clinical features. It is characterized clinically by retarded growth, intellectual disability, typical facial features, broad and short thumbs, and halluces (3,4).

Cardiac defects in RTS include atrial septal defect, ventricular septal defect, PDA, coarctation of the aorta, pulmonic stenosis, bicuspid aortic valve, pseudo-truncus, aortic stenosis, dextrocardia, vascular rings, conduction disorders and occasionally, hypoplastic left heart (5). In 1995, a study was performed to evaluate the frequency and type of cardiac defects and their significance in RTS (10). Among the 138 patients in that study, 40 (32.6%) had a known cardiac abnormality; 27 patients had single defects including atrial septal defect, VSD, PDA, coarctation of the aorta, pulmonic stenosis, or bicuspid aortic valve; 16 patients had complex congenital heart defects or two or more abnormalities; and 5 patients had conduction abnormalities (10). Little to no literature is available to establish an association between RTS and DCM; the purpose of the present case report is to provide insight into this condition. RTS is linked to a range of cardiac abnormalities, including DCM. The variability in cardiac involvement requires careful assessment and the personalized management of each patient.

A limitation of the present study is the lack of molecular confirmation. Genetic testing for CREBBP and EP300 mutations could not be performed due to resource constraints in the authors' setting. Therefore, the diagnosis was established clinically according to the Lacombe *et al* (8) consensus criteria. This limits the ability to directly associate the patient's phenotype with specific genotypic findings. Additional research is crucial in order to elucidate the mechanisms connecting RTS to cardiac dysfunction and to develop effective treatment approaches.

In conclusion, RTS is a rare condition with diverse clinical features, requiring further studies to refine diagnostic guidelines. The present case report aimed to update the RTS guidelines, particularly its association with heart failure and DCM, which has not been previously published. RTS should be considered in patients with broad thumbs, halluces and characteristic facial features, as it may be underreported in Pakistan, with only one documented case.

Following the diagnosis of RTS, comprehensive evaluations, including cardiac, ophthalmic, renal, endocrine, orthopedic and tumor screening, are essential for early intervention. The case described herein is unique due to the rare coexistence of RTS and severe DCM (LVEF 20%), a condition typically linked to congenital heart defects rather than cardiomyopathy. Potential mechanisms include genetic predisposition, chronic volume overload, and metabolic dysfunction.

Long-term management involves heart failure therapy, with adherence challenges due to intellectual disability, requiring caregiver education and close monitoring. A multidisciplinary approach is crucial, emphasizing the need for further research into the genetic and metabolic links of RTS with cardiomyopathy.

## Figures and Tables

**Table I:** Summary of reported cases of cardiac anomalies and dilated cardiomyopathy in patients with syndromes identified in the literature.

Study	Age of the patients	Sex	Cardiac anomalies	Dilated cardiomyopathy	Outcome	(Refs.)
Case 1	5 years	Male	PDA (4 mm)	Not specified	Managed conservatively	(11)
Cases2	2 years	Female	Tricuspid atresia, pulmonary atresia	Present	Surgical intervention required	(9)
Case 3	8 years	Male	VSD, ASD	Present	Ongoing management for heart failure	(12)
Case 4	6 years	Male	Multiple congenital defects	Not specified	Complications from malignancy	(13)
Case 5	10 years	Female	Aortic coarctation	Present	Surgical repair performed	(14)
Current case	32 years	Male	None	Present (LVEF 20%)	Responded well to IV diuretics; referred for cardiology	Present study

PDA, patent ductus arteriosus; VSD, ventricular septal defect; ASD, atrial septal defect; LVEF, left ventricular ejection fraction.

## Data Availability

The data generated in the present study may be requested from the corresponding author.

## References

[1] Michail J, Matsoukas J, Theodorou S (1957). Arched, clubbed thumb in strong abduction-extension & other concomitant symptoms. Rev Chir Orthop Reparatrice Appar Mot.

[2] Rubinstein JH, Taybi H (1963). Broad thumbs and toes and facial abnormalities. A possible mental retardation syndrome. Am J Dis Child.

[3] Bartsch O, Labonté J, Albrecht B, Wieczorek D, Lechno S, Zechner U, Haaf T (2020). Two patients with EP300 mutations and facial dysmorphism different from the classic Rubinstein-Taybi syndrome. Am J Med Genet A.

[4] Negri G, Magini P, Milani D, Colapietro P, Rusconi D, Scarano E, Bonati MT, Priolo M, Crippa M, Mazzanti L (2016). From whole gene deletion to point mutations of EP300-positive rubinstein-taybi patients: New insights into the mutational spectrum and peculiar clinical hallmarks. Hum Mutat.

[5] Milani D, Manzoni FM, Pezzani L, Ajmone P, Gervasini C, Menni F, Esposito S (2015). Rubinstein-Taybi syndrome: Clinical features, genetic basis, diagnosis, and management. Ital J Pediatr.

[6] López M, García-Oguiza A, Armstrong J, García-Cobaleda I, García-Miñaur S, Santos-Simarro F, Seidel V, Domínguez-Garrido E (2018). Rubinstein-Taybi 2 associated to novel EP300 mutations: Deepening the clinical and genetic spectrum. BMC Med Genet.

[7] Kamenarova K, Simeonov E, Tzveova R, Dacheva D, Penkov M, Kremensky I, Perenovska P, Mitev V, Kaneva R (2016). Identification of a novel de novo mutation of CREBBP in a patient with Rubinstein-Taybi syndrome by targeted next-generation sequencing: A case report. Hum Pathol.

[8] Lacombe D, Bloch-Zupan A, Bredrup C, Cooper EB, Houge SD, García-Miñaúr S, Kayserili H, Larizza L, Lopez Gonzalez V, Menke LA (2024). Diagnosis and management in Rubinstein-Taybi syndrome: First international consensus statement. J Med Genet.

[9] Loomba RS, Geddes G (2015). Tricuspid atresia and pulmonary atresia in a child with Rubinstein-Taybi syndrome. Ann Pediatr Cardiol.

[10] Stevens CA, Bhakta MG (1995). Cardiac abnormalities in the Rubinstein-Taybi syndrome. Am J Med Genet.

[11] Desai K, Taksande A, Meshram R, Jain A (2023). Beaked nose with syndactyly: A rare case of Rubenstein-Taybi syndrome. Med Sci.

[12] Scaglia F, Towbin JA, Craigen WJ, Belmont JW, Smith EO, Neish SR, Ware SM, Hunter JV, Fernbach SD, Vladutiu GD (2004). Clinical spectrum, morbidity, and mortality in 113 pediatric patients with mitochondrial disease. Paediatrics.

[13] Taylor MD, Mainprize TG, Rutka JT, Becker L, Bayani J, Drake JM (2001). Medulloblastoma in a child with Rubenstein-Taybi syndrome: Case report and review of the literature. Pediatr Neurosurg.

[14] van Voorden AJ, Keijser R, Veenboer GJM, Lopes Cardozo SA, Diek D, Vlaardingerbroek JA, van Dijk M, Ris-Stalpers C, van Pelt AMM, Afink GB (2023). EP300 facilitates human trophoblast stem cell differentiation. Proc Natl Acad Sci USA.

